# 3D printed microfluidic valve on PCB for flow control applications using liquid metal

**DOI:** 10.1007/s10544-024-00697-z

**Published:** 2024-01-30

**Authors:** Ahmed Hamza, Anagha Navale, Qingchuan Song, Sagar Bhagwat, Frederik Kotz-Helmer, Pegah Pezeshkpour, Bastian E. Rapp

**Affiliations:** Laboratory of Process Technology, NeptunLab, Department of Microsystem Engineering (IMTEK), University of Freiburg, 79110 Freiburg, Germany

**Keywords:** 3d printing, Microfluidic valves, Pcb, Liquid metals, Electrochemical actuation

## Abstract

Direct 3D printing of active microfluidic elements on PCB substrates enables high-speed fabrication of stand-alone microdevices for a variety of health and energy applications. Microvalves are key components of microfluidic devices and liquid metal (LM) microvalves exhibit promising flow control in microsystems integrated with PCBs. In this paper, we demonstrate LM microvalves directly 3D printed on PCB using advanced digital light processing (DLP). Electrodes on PCB are coated by carbon ink to prevent alloying between gallium-based LM plug and copper electrodes. We used DLP 3D printers with in-house developed acrylic-based resins, Isobornyl Acrylate, and Diurethane Dimethacrylate (DUDMA) and functionalized PCB surface with acrylic-based resin for strong bonding. Valving seats are printed in a 3D caterpillar geometry with chamber diameter of 700 µm. We successfully printed channels and nozzles down to 90 µm. Aiming for microvalves for low-power applications, we applied square-wave voltage of 2 V_pp_ at a range of frequencies between 5 to 35 Hz. The results show precise control of the bistable valving mechanism based on electrochemical actuation of LMs.

**Supplementary Information:**

The online version contains supplementary material available at 10.1007/s10544-024-00697-z.

## Introduction

Microfluidics applies the power of miniaturization to create new devices which outperform their larger predecessors in several sectors such as health and energy. The fluidic manipulation in microfluidic devices relies on fluidic microvalves, as one of the fundamental components in any fluidic system. The performance of such valves in flow control is a key step in microfluidic devices for applications in the Life Sciences (Streets and Huang 2013; Studer et al. 2003). Many microvalves are the miniaturized form of their conventional counterparts, which are either complex in structure or require complex assembly and integration with other components in the microfluidic system. While the portable microfluidic devices are fully functional, the integration of fluidic control components, in particular valves and tubing, with the microchip is not free of complexity (Zhu et al. 2023). Moreover, the microvalves materials and their fabrication techniques bring some limitations when it comes to the applications in Life Sciences. Several approaches addressed the challenges of complex etching process of fabrication of microsystems from glass or silicon or soft lithography of microfluidic chips from polydimethylsiloxane (PDMS) (Au et al. 2016; Kotz et al. 2019). In addition to being limited to planar designs acting as passive microdevices, tediously slow fabrication process in soft lithography, and the interface layer required for large number of connectors and wells do not meet industry standards in point-of-care or life sciences applications. However, development of miniaturized devices benefits from 3D printing in many aspects from speeding up the fabrication of actual 3D structures containing non-planar elements in compact microdevices. Recent research on fabrication techniques for microfluidic elements shows the paradigm shift towards 3D printing and replacing PDMS soft lithography (Bhattacharjee et al. 2016; Boaks et al. 2023; Hinnen et al. 2023; Waheed et al. 2016; Diehm et al. 2021; Goralczyk et al. 2022; Hinnen et al. 2023; Rogers et al. 2015; Weisgrab et al. 2019). Based on several valving mechanisms, common valves in microfluidics are thermopneumatic valves (Rich and Wise 2003), membrane-type valves (Lee et al. 2018), electrostatic microvalves (Desai et al. 2012), hydrogel microvalves (Wang et al. 2005) or electromagnetic microvalves (Bintoro et al. 2005). In electrochemical-flow control valves, the all-in-one microfluidic device is integrated with the printed circuit boards (PCBs). Thus, Lab-on-PCBs have been developed as stand-alone devices like biosensors and diagnosis systems (Zhao et al. 2020). Prior to merging of 3D printing and microfluidics, the integration of microfluidic elements with PCBs has been mostly through clamping, taping, adhesives, or punching holes and screwing the two parts together (Baek et al. 2023; Thanh et al. 2022; Wan et al. 2019). Apart from prototypes and lab studies, the development of microfluidic system with PCB towards more commercialized products necessitates proper and durable bonding. This needs the integration of the microfluidic valve from a 3D-printable resin to bond properly with the outermost layer of PCB patterned with electrodes (Perdigones 2021; Thanh et al. 2022). Recently, liquid metal (LM) bistable valves received more attention owing to unique properties of LMs (Bhagwat et al. 2022; Daeneke et al. 2018; Eaker and Dickey 2016; Gough et al. 2014). However, a common challenge in LM valves and actuators is the alloying of elements in the LM, in particular Gallium, with common metal electrodes. As a result of alloying, the LM plug, i.e. the core of valving mechanism, adheres to the surface of electrodes which leads to presence of alloying residue and consequently limiting the entire actuation. Several approaches are presented in literature to overcome this challenge. Sivan et al. introduced LM marbles by employing WO_3_ and polytetrafluoroethylene (PTFE) to prevent adhering of LMs to metal surfaces (Sivan et al. 2013). Another approach was adding a protective layer coated on the electrodes to isolate the metal surface from LMs. Our group previously investigated alloying of several metal electrodes in contact with LM, and have shown Polypyrrole coated gold electrodes to have LM alloying barrier properties (Bhagwat et al. 2023). Another surface coating to protect electrodes from sticking of LMs is a layer of carbon. Oh et al. presented spraying carbon nanotubes (CNTs) on an Ag thin film surface as a barrier for reliable and stretchable electronics (Oh et al. 2018). Combining the alloying barrier properties of a commercially available carbon ink with a simple process like screen printing would allow for scalability.

Here, we present a simple procedure of direct 3D printing of microvalve on a PCB substrate, patterned with carbon-coated Cu electrodes. The valving mechanism is based on the electrochemical actuation of LMs in caterpillar-shaped chambers. While all the multi-layer valves presented in literature so far do the flow control in precise manner, minimizing layers significantly simplifies the fabrication and actuation process. Hence, our work focuses on having a single-layer bistable valve through actuation of a Galinstan LM plug. Moreover, our one-step direct 3D printing of the microchip saves the time for tedious fabrication steps followed by alignment and bonding procedures. Our stand-alone device consists of a directly 3D-printed microvalve on a PCB with the most advanced additive manufacturing technique to date.

## Experimental

### Electrodes fabrication

**Fig. 1:**
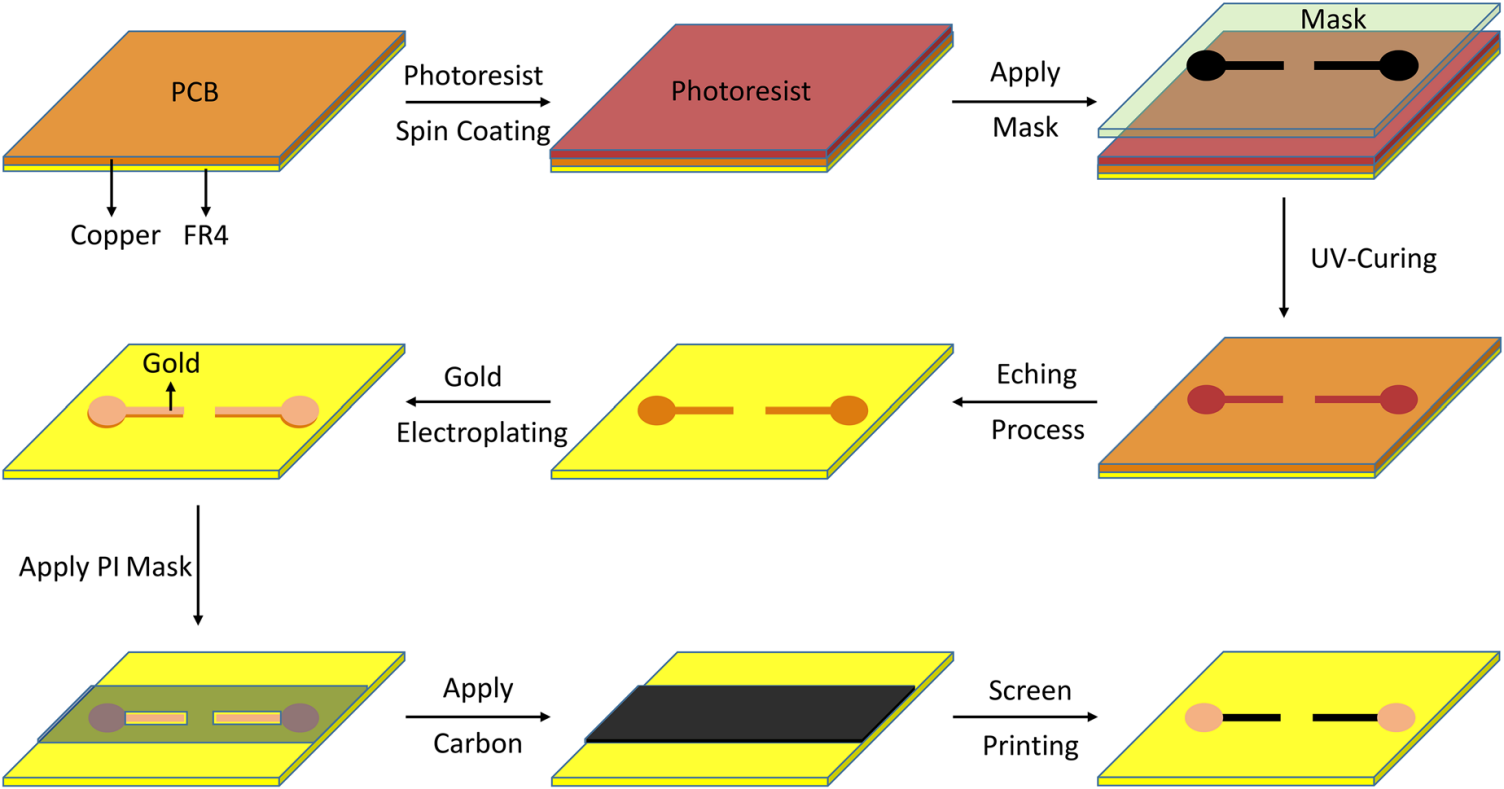
The fabrication process for carbon-coated copper electrode on PCB

The substrate on which the microvalve is directly 3D printed is an FR4 PCB with one-sided copper layer (Bungard, Germany). Photolithography process was used to pattern the copper electrodes on the PCB. The process is explained elsewhere but briefly includes the following process as shown in Fig. 1. First, AZ1518 (Microchemicals, Germany) photoresist was spin coated on the PCB. Following a soft baking step on a heating plate at 107 °C for 1.5 min, mask-based lithography was carried out. The mask including electrodes pattern was designed in Autodesk Inventor and placed on top of the PCB. The mask was printed on a transparent plastic foil (Soennecken eG, Germany) with the printer TASKalfa 4052ci (STREIT SERVICE & SOLUTION GMBH & CO. KG, Germany). The PCB was UV-cured at 415 nm for 1 min prior to the post-exposure baking step on a heating plate at 107°C for 1.5 min. To remove the excess photoresist, the PCB was immersed in AZ351B developer diluted 1:4 (vol/vol) in water. Finally, the copper layer was etched using 25 wt% Ammonium peroxodisulfate (Sigma Aldrich, Germany) in water. The solution was mixed on a magnetic stirrer at 400 rpm and 50 °C. The PCB was in the solution for 5–10 min until all the unexposed copper was etched. To protect the copper electrodes from corrosion, a gold layer was electroplated on top of copper with 2 µm targeted thickness. A gold electrolyte Auroblex 3202 (Blend GMBH, Germany) solution was used at 55 °C and 20 mA/cm^2^ to create the desired thickness. To prevent alloying between Galinstan and the Gold-plated copper electrode, a protective carbon layer was added on top. Based on the screen printing technique, a polyimide tape (Elegoo Inc, China) with a thickness of 25 µm was used as the shadow mask and was placed on top of the PCB substrate. The mask pattern was designed using Autodesk Inventor software and was exported as a DXF file to be used for laser machining (Protolaser U4, LPKF). The laser beam was set at 15 µm, 25 kHz frequency, 1.5 W power, 130 mm/s Mark speed, and 5 repetitions to cut the polyimide tape. Conductive carbon ink, SD 2842 HAL (ELPEPCB, Germany), was applied on the mask using a spatula. Following this step, the PCB patterned with carbon-coated copper electrodes was annealed in a 150 °C oven for 45 min. The final step was taken by removing the shadow mask.

### Resin preparation

An acrylic-based resin used consisted of 49.84 wt% of Isobornyl Acrylate and 49.84 wt% of Diurethane Dimethacrylate (DUDMA) which were mixed in a bottle using the Sonorex Da300 ultrasonic bath (Bandelin electronic, Germany) at 50 °C for 20 min. The photoinitiator and absorber, 0.3 wt% of Diphenyl(2,4,6-trimethylbenzoyl)phosphine oxide (TPO) and 0.03 wt% of Sudan Orange (Sigma Aldrich, Germany), were added to the mixture in the ultrasonic bath for another 5 min.

### 3D printing on PCB and PCB functionalization

**Fig. 2:**
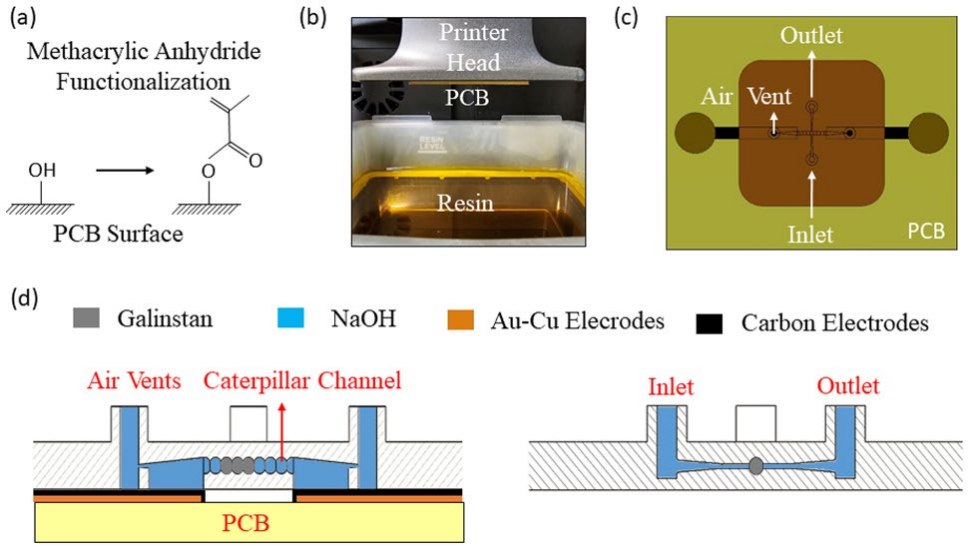
**a** PCB surface after functionalization. **b** 3D printing of the microfluidic chip on the functionalized PCB. **c** Schematic diagram of the microfluidic chip on PCB. **d** Schematic cross-section views of the microfluidic chip on PCB

To provide the bonding between 3D printed microchip from acrylic-based resin and the PCB, PCB surface was functionalized with an acrylic-based solution as shown in Fig. 2a. A 29.19 wt% methacrylic anhydride was mixed with 70.81 wt% of Dimethylaminopyridine (DMAP) (Sigma Aldrich, Germany) in an ultrasonic-bath at 50 °C for 10 min. The PCB was immersed in the solution for 24 h and was rinsed in acetone afterwards. An FTIR spectrometer (Frontier 100 MIR-FTIR, Perkin Elmer, Germany) was used to characterize the PCB surface. A tensile test was also conducted using Zwick/Z010 machine (Germany) to examine the adhesion of the microchip to the PCB.

The microfluidic chip was designed using Autodesk Inventor software. As shown in Fig. 2c-d, the inlet of the chip is connected to a caterpillar shaped chamber that would be filled with LM plugs. The outlet of the chip was the controlled fluid from the system.

Before printing the microfluidic chip, an alignment design (Fig. S1) was printed on the printer head to precisely align the chip with the electrodes on PCB. Following the functionalization step of the PCB, it was cleaned with acetone and attached to the printer head using a double-sided tape (Fig. 2b). To directly print the microvalve chip on PCB, we used Asiga Max X DLP printer (Asiga, Alexandria, NSW, Australia) with a pixel resolution of 27 µm and a build volume of 51.8 × 29.2 x 75.0 mm^3^. The zero position of the printer head was re-adjusted with 2 mm offset to compensate for the PCB thickness. As shown in Table 1, the printing parameters were updated during the printing process. The exposure time of the first layer (burn-in) was set to 20 s to obtain proper adhesion and the light intensity was set at a lower value, 9 mW/cm^2^, to avoid curing the layer covering the electrodes. The exposure time and the light intensity for the remaining layers were set at a faster pace of 3 s and 30 mW/cm^2^ respectively. After the printing step was completed, the channels were developed in isopropanol followed by removing the excess uncured resin through a pressure-driven pump (Fluigent, Germany) at 300 mbar for 5 min.

**Table 1:** Printing Parameters of the microfluidic chip

Parameter	Burn-in	Layer 2–5	Other layers
Temperature [°C]	25	25	25
Light Intensity [mW/cm^2^]	9	9	30
Exposure Time [s]	20	3	3
Z Compensation [mm]	0.708	0.216	0.446

### LM valve implementation and characterization

Both PCB and the microfluidic chip were plasma treated (Plasma Electronic, Germany) for 5 min at 0.3 mbar to activate the surfaces in the flow channels, valving chambers, and electrodes. Before injecting Galinstan (Strategic Elements, Germany) into the chambers, 1M NaOH was injected into the microfluidic channels using a syringe to break the oxide skin on the Galinstan. Galinstan was then injected into the channel filling three chambers in Fig. 2d. Since the valving mechanism is based on the electrochemical actuation of Galinstan in the chambers, the characterization of the valve actuation was performed. A waveform generator 33220A (Agilent, Germany) provided square-wave signals with peak-to-peak voltage of 0.5 to 2 V_pp_ at frequencies ranging from 1 to 35 Hz. The valve response time was analyzed with slow motion videos conducted using Shotcut software. The hydrostatic pressure method was used to determine the amount of pressure the valve would withstand.

## Results and discussion

### Effect of the functionalization on bonding

**Fig. 3:**
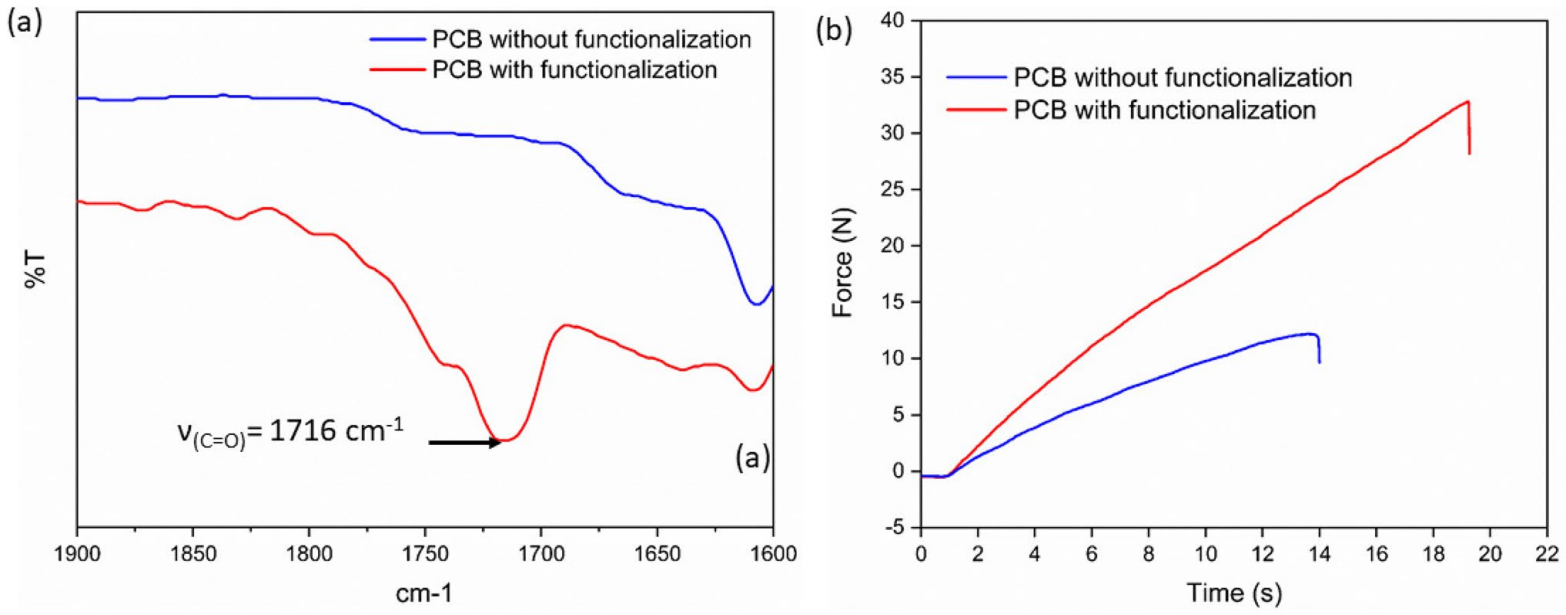
Functionalization effect on PCB. **a** FTIR assessment of the pristine (blue) and the surface-modified PCB (red) showing the presence of the characteristic peaks of the formation of esters. **b** Tensile test of the pristine (blue) and surface-modified PCB (blue) confirming enhancement of adhesion after surface modification

Most PCBs that are available on the market consist of phenolic resin-based polymers with fillers. These phenolic resins typically possess active hydroxyl groups, allowing for potential esterification reactions. The significance lies in the fact that surface modification through esterification reactions enables the attachment of the monomer groups, here acrylates and methacrylates, to the surface. These groups have the capability to copolymerize with the monomers in the resin which results in a strong adhesion of the printed structure to the PCB. To verify this, we conducted an experiment by immersing the PCBs in a mixture of methacrylic anhydride and DMAP. Subsequently, we analyzed the changes in the infrared absorption of the PCBs before and after the process using FTIR. The FTIR results, depicted in Fig. 3a, revealed a prominent absorption peak near 1716 cm^−1^ after the immersion in methacrylic anhydride. In the infrared spectrum, this absorption peak is indicative of the presence of a strong ester C = O bond, which is characteristic of the methacrylate ester structure. To confirm the FTIR results, a tensile test was conducted with a dog-bone shape specimen attached to the chip. The holder to the chip has a dimension of 4 × 4 mm^2^ and the tensile test speed was 1 mm/min. As shown in Fig. 3b, the PCB without functionalization delaminated at a force of 13 N, whereas the PCB with functionalization delaminated at a force of 32 N. Therefore, these findings confirm the successful modification of the PCB surface.

### Liquid metal valving mechanism

**Fig. 4:**
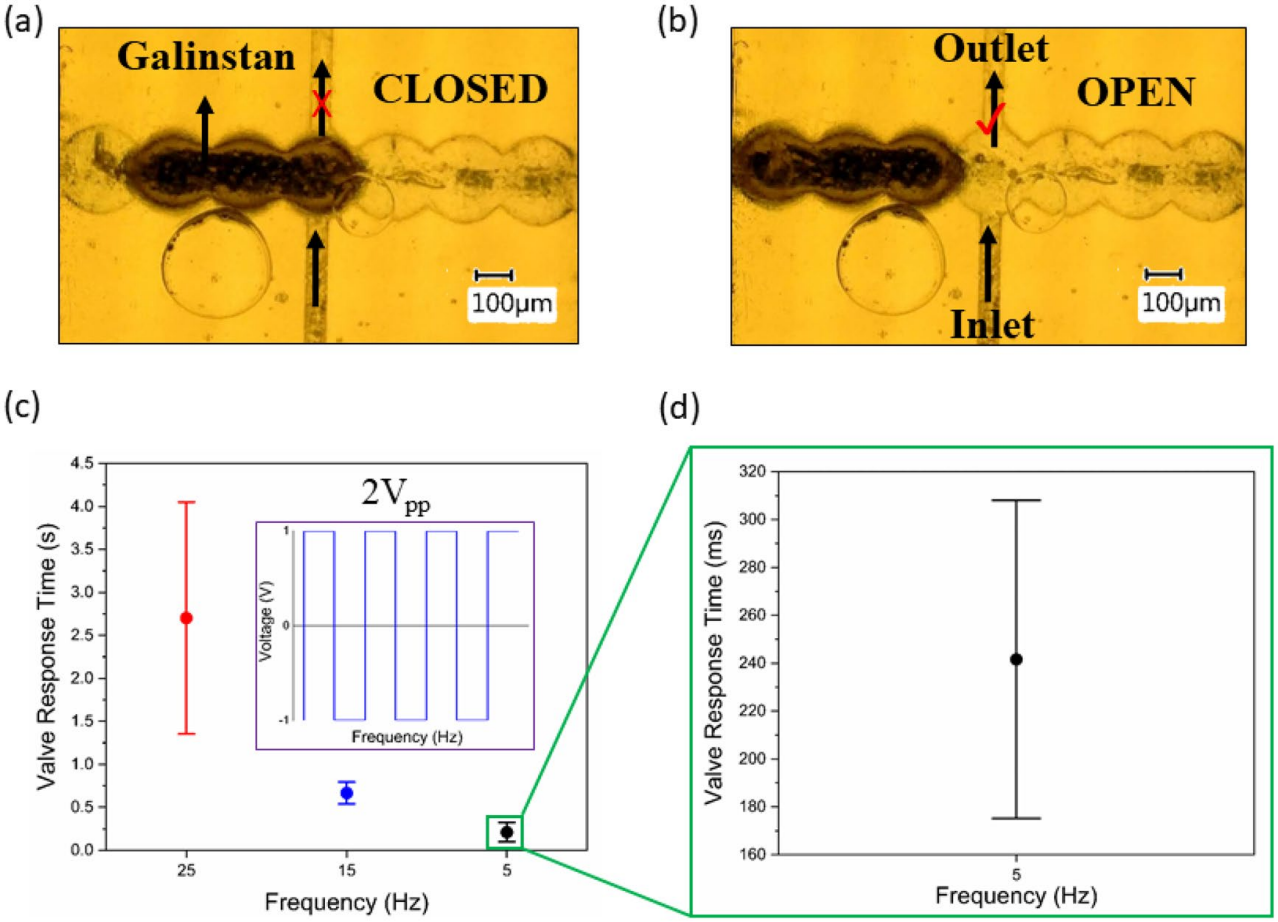
**a** The CLOSED-State of the LM valve. **b** The OPEN-State of the LM valve. **c** The valve response time in seconds of 6 tests at different frequencies. **d** The valve response time in milliseconds at 5 Hz frequency

The valving mechanism of the present microvalve, directly 3D printed on a PCB, is based on the electrochemical actuation of LM, i.e. Galinstan plugs. As discussed, Galinstan is a liquid metal alloy and is composed of gallium, indium, and tin (68.5 wt% Ga, 21 wt% In, 10 wt% Sn; melting point: 13.2 °C) (Daeneke et al. 2018). Despite its attracting properties as a liquid metal at room temperature, including high electrical conductivity (around 3.4 × 10^6^ S m^−1^, about 17 times lower than copper), low viscosity (about twice the viscosity of water), high surface tension (around 600–700 mN m^−1^), extremely low toxicity, and negligible vapor pressures (< 10^−6^ Pa) (Khoshmanesh et al. 2017), the oxide skin hinders its flow in microchannels. A nanometer-thick oxide skin is formed on its surface when exposed to air which affects its conductivity and also surface adhesion. Manipulating Galinstan in a microfluidic channel requires an electrolyte that can, firstly, remove this oxide skin and, secondly, provides enough ion conductivity to actuate the Galinstan plug. Based on previous experience (Bhagwat et al. 2022), NaOH meets both criteria. Following filling the channels and chambers with NaOH and injecting the Galinstan to fill three chambers, electric potential is applied to the electrodes. The Galinstan plug starts moving under the applied electric field (Fig. 4a-b). Upon clearing the chamber intersecting with the flow channel (the vertical channel from inlet to outlet), the valve goes from CLOSED state to OPEN state. We defined the CLOSED-state of the valve when Galinstan plug blocks the vertical channel. Under certain voltage, the plug moves by one chamber, clears the vertical channel and the microvalve switches to the OPEN-state. We observed that two parameters play roles mainly in maintaining the microvalve in bistable modes: the applied electric potential and the geometry of the valve containing LM plugs.

####  Modulated voltage for LM actuation

One common challenge in the electrochemical actuation of LMs in microchannels filled with electrolytes is the hydrolysis and subsequent gas formation the channels. The bubbles not only result in blocking the microchannels, but also create an insulating layer between the electrodes and the LM plug, hindering its actuation and displacement. The required voltage to move the LM plug depends on several factors including the plug size, electrolyte concentration, channel dimensions, and voltage type and range. Aiming for low power applications, our experiments did not show significant changes in the results changing the electrolyte concentration. Therefore, we focused mainly on looking into the effect of applied voltages and the architecture of the valve. For lower voltage range and more precise control over the plug motion in the chambers, we selected square-wave over DC voltage. This is mainly due to the fact that an aqueous NaOH solution imposes hydrolysis limitations under applied voltages approximately at 1.2 V. When applying a DC voltage with constant current, the voltage required to move Galinstan is more than 4 V which leads to instantly the formation of air bubbles. Moreover, Gough et al. had presented the effect of implementing low AC voltages to successfully break the continuously regenerating oxide skin, in comparison to high DC voltages which eventually result in slow LM plug movement due to the oxide skin (Gough et al. 2014). Therefore, we selected the square-wave signal over DC voltages. The actuation of Galinstan plugs benefits from such modulated voltage, which does not apply a constant current to the system and provides switching polarity at constant frequency intervals. A waveform generator (Fig. S2) generates the modulated signals and is connected to the electrodes through two wires soldered on the gold pads. In this work, we investigated the voltage amplitude and frequency. Initially, we observed that changing the voltage from 0.5 V_pp_ to 1.5 V_pp_ was not strong enough to move the plug. However, at 2 V_pp_, the Galinstan plug started moving. Moreover, we found the frequency should not be too high or too low, hence there is a frequency interval to move the plug. We characterized the valve for a range of frequencies changing them from 1 to 35 Hz at this specified voltage (2 V_pp_). For frequencies lower than 5 Hz or higher than 25 Hz, the plug vibrates only in place (actuation video S 1) and thus does not provide valving capability. We achieved a reproducible and stable actuation between 5—25 Hz. Figure 4c presents the characterization of the microvalve response time implemented at three frequencies (5, 15 and 25 Hz), the valve response time was statistically analyzed with 6 test per each frequency value between the OPEN and CLOSED states. At 25 Hz the average response time is approximately 2700 ms with high error margin (actuation video S 2), as the plug moves too fast preferring to remain in the initial state. As going down to 15 Hz (actuation video S 3), the average response time improved to approximately 650 ms. The best response time with reproducible actuation in our observation was 250 ms which happened at 5 Hz (actuation video S 4), as shown in Fig. 4d. This resulted response time at 5 Hz showed fast actuation of the LM plug compared to different valving mechanisms (Díaz-González et al. 2016; Si et al. 2017).

#### 3D Geometry for the LM microvalve

Given the nature of Galinstan, its surface tension and actuation, another factor plays a key role on a LM microvalve, i.e., the valve’s geometric architecture. Due to the high surface tension of Galinstan, and its coalescence tendency, Galinstan plugs would prefer to remain connected while moving in uneven channels. As shown in Fig. 4c, a caterpillar design allows the LM to fill the entire chamber in the vicinity of the inlet and the outlet channels. The air vents on the right and left sides of the valve (Fig. 2c-d) are intended for three purposes: a) to give the liquid enough space to move while actuating the LM without inducing pressure in the system; b) to provide a larger contact area with the electrodes for enough electric field; and c) to move away any air bubble generated on the electrodes. To control the direction of the liquid towards the outlet channel, the dimensions of the nozzles to the air vents were set to half of the inlet/outlet channels.

#### Valve-Pressure test

The valve was tested using the hydrostatic pressure method. A beaker with a tube was placed at the same level as the chip located. NaOH solution with a food color was poured into the beaker and the pressure applied to the chip was calculated. We observed that the valve did hold pressure until 2 mbar, then started leaking from the side channel. The pressure the valve can hold would be further increased by reducing the channel size by using a higher resolution printer.

## Conclusion and outlook

A microfluidic valving chip with a nozzle size of 90 µm was successfully 3D printed on a PCB substrate using Asiga Max printer. A reliable adhesion was obtained using the proper functionalization and the results showed holding 32 N force required to delaminate the chip from the PCB. The microfluidic bistable valve worked reliably based on liquid metal electrochemical actuation under a low square voltage of 2 V_pp_ fitting low power applications. The valve can hold up to 2 mbar pressure and several frequencies have been tested to actuate the LM for the OPEN/CLOSED states. The fastest response time observed was 200 ms at 5 Hz. The response time and the threshold of valving pressure could be further investigated by overcoming the hydrolysis challenges and also higher printing resolution for narrowing down the flow channels. The presented LM microvalve in this work well integrates with microsystems for numerous applications in the biomedical and lab-on-chip with no pneumatic pumps involved.

### Electronic supplementary material

Below is the link to the electronic supplementary material.

Supplementary Material 1

Supplementary Material 2

Supplementary Material 3

Supplementary Material 4

Supplementary Material 5

## Data Availability

The data that support the findings of this study are available from the corresponding author upon reasonable request.
