# Supplementary material for: 3D printed microfluidic valve on PCB for flow control applications using liquid metal
Source: Biomed Microdevices. 2024 Jan 30;26(2):14. doi: 10.1007/s10544-024-00697-z (PMC10827904; doi:10.1007/s10544-024-00697-z)
Supplement: Supplementary file 1 — Supplementary Material 1 [file 10544_2024_697_MOESM1_ESM.docx]

# Supplementary Information

3D printed Microfluidic Valve on PCB for Flow Control Applications using Liquid Metal

Ahmed Hamza [
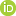
](https://orcid.org/0009-0009-1518-5582), Anagha Navale, Qingchuan Song [
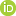
](https://orcid.org/0000-0003-2548-8009), Sagar Bhagwat [
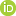
](https://orcid.org/0000-0002-9295-0606), Pegah Pezeshkpour* [
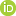
](https://orcid.org/0000-0003-2583-1532) and Bastian E. Rapp [
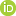
](https://orcid.org/0000-0002-3955-0291)

Laboratory of Process Technology, NeptunLab, Department of Microsystem Engineering (IMTEK), University of Freiburg, 79110 Freiburg im Breisgau, Germany.

*Corresponding author. E-mail: Pegah.Pezeshkpour@neptunlab.org

# Supplementary Information

Aligning the PCB on the printer head could be challenging due to the need a precise adjustment of the PCB with the design during printing. After calibrating the Z-position of the Printer, the center of the design would be shifted from the center of the printer head. To solve this problem, a calibration design as shown in Fig. S 1, was printed first on the printer head to set the borders of the printable area and to align the electrodes in the middle.


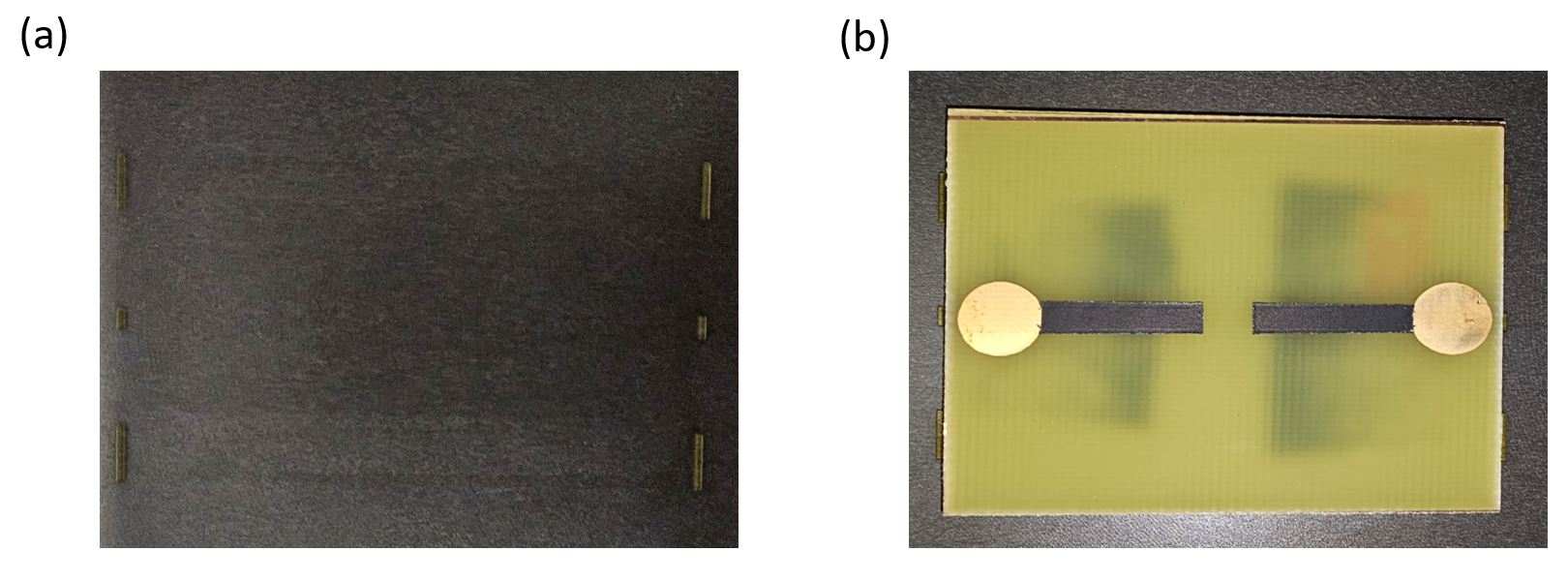


Fig. S 1 **a** Alignment design printed on the printer head. **b** The fixed PCB on the printer head

As shown in Fig. S 2a, a wave generator was used to supply a square wave pattern to move the Galinstan valve. Fig. S 2b shows the 2 cross-section views of the microfluidic chip design and its connection with the carbon electrodes.


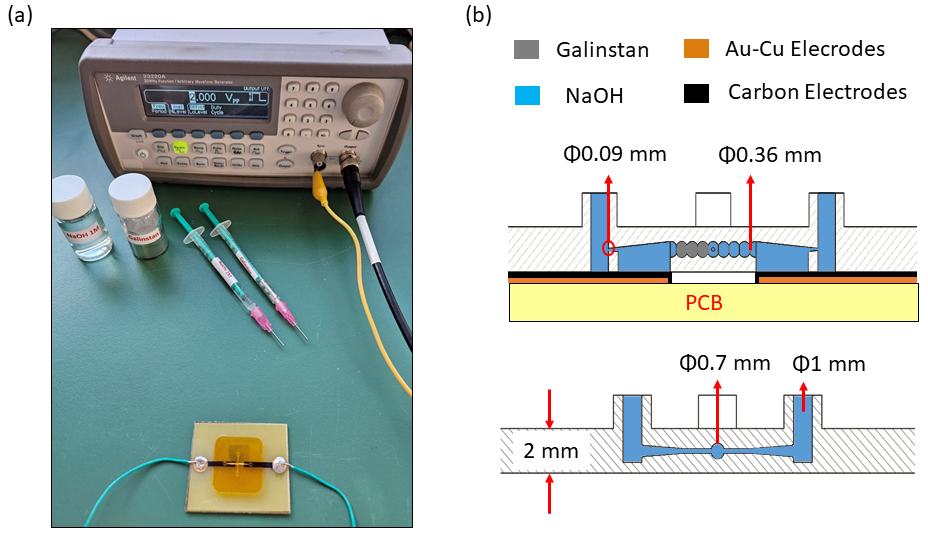


Waveform Generator

Fig. S 2 **a** The experimental setup used for the LM valve. **b** Schematic cross-section views of the microfluidic chip on PCB
